# Anti-PD-L1 immunoconjugates for cancer therapy: Are available antibodies good carriers for toxic payload delivering?

**DOI:** 10.3389/fphar.2022.972046

**Published:** 2022-08-16

**Authors:** Andrea Zanello, Massimo Bortolotti, Stefania Maiello, Andrea Bolognesi, Letizia Polito

**Affiliations:** Department of Experimental, Diagnostic and Specialty Medicine-DIMES, Alma Mater Studiorum, University of Bologna, Bologna, Italy

**Keywords:** PD-1, PD-L1, immunotherapy, immunoconjugates, cancer therapy

## Abstract

Immune checkpoint mechanisms are important molecular cell systems that maintain tolerance toward autoantigens in order to prevent immunity-mediated accidental damage. It is well known that cancer cells may exploit these molecular and cellular mechanisms to escape recognition and elimination by immune cells. Programmed cell death protein-1 (PD-1) and its natural ligand programmed cell death ligand-1 (PD-L1) form the PD-L1/PD-1 axis, a well-known immune checkpoint mechanism, which is considered an interesting target in cancer immunotherapy. In fact, the expression of PD-L1 was found in various solid malignancies and the overactivation of PD-L1/PD-1 axis results in a poor patient survival rate. Breaking PD-L1/PD-1 axis, by blocking either the cancer side or the immune side of the axis, is currently used as anti-cancer strategy to re-establish a tumor-specific immune response. For this purpose, several blocking antibodies are now available. To date, three anti-PD-L1 antibodies have been approved by the FDA, namely atezolizumab, durvalumab and avelumab. The main advantages of anti-PD-L1 antibodies arise from the overexpression of PD-L1 antigen by a high number of tumor cells, also deriving from different tissues; this makes anti-PD-L1 antibodies potential pan-specific anti-cancer molecules. Despite the good results reported in clinical trials with anti-PD-L1 antibodies, there is a significant number of patients that do not respond to the therapy. In fact, it should be considered that, in some neoplastic patients, reduced or absent infiltration of cytotoxic T cells and natural killer cells in the tumor microenvironment or presence of other immunosuppressive molecules make immunotherapy with anti-PD-L1 blocking antibodies less effective. A strategy to improve the efficacy of antibodies is to use them as carriers for toxic payloads (toxins, drugs, enzymes, radionuclides, etc.) to form immunoconjugates. Several immunoconjugates have been already approved by FDA for treatment of malignancies. In this review, we focused on PD-L1 targeting antibodies utilized as carrier to construct immunoconjugates for the potential elimination of neoplastic cells, expressing PD-L1. A complete examination of the literature regarding anti-PD-L1 immunoconjugates is here reported, describing the results obtained *in vitro* and *in vivo*. The real potential of anti-PD-L1 antibodies as carriers for toxic payload delivery is considered and extensively discussed.

## Introduction

Immune checkpoints (ICs), including programmed cell death protein-1 (PD-1), programmed cell death ligand-1 (PD-L1) and cytotoxic T lymphocyte–associated antigen-4 (CTLA-4), are a class of immune-modulating proteins involved in the negative regulation of the immune system. PD-1 and CTLA-4 provide immunosuppressive signals when bound to PD-L1 and CD80/86, respectively. These immunosuppressive molecules prevent cytotoxic effectors to harm self-cells, thus maintaining peripheral tolerance, impeding autoimmunity in physiological conditions and avoiding chronically activation of immune response.

In almost all solid cancers, the tumor microenvironment is characterized by an immunosuppressive milieu, due to the secretion of specific molecules (e.g., IL-10 and IFN-γ) (Sarvaria et al., 2017; Labani-Motlagh et al., 2020) and the exploitation of IC functions, allowing cancer cells to escape immune surveillance, thus leading to cancer progression and patient prognosis worsening.

Immune checkpoint inhibitors (ICIs) are a class of anti-cancer antibodies that block PD-1, PD-L1 or CTLA-4. This blockade induces the T-cell reinvigoration and the cytotoxic T-cell activity against tumor cells, therefore forcing the balance of the immune system to shift from a passive to an active anti-tumor state. However, despite the encouraging outcomes in responder patients, the percentage of patients estimated to respond to ICI therapy is only 13% (Haslam and Prasad, 2019). This is because a relevant group of patients experience innate or acquired resistance to ICI therapy due to cancer cells’ adaptative mechanism in antigen formation and/or presentation (e.g., impaired cell surface expression of MHC class I) and mutations in immune effector signaling pathway (e.g., JAK1/JAK2 or B2M) leading to insufficient anti-tumor T-cell generation or inadequate T-cell effector function (Syn et al., 2017; Jenkins et al., 2018). To note, PD-L1 mutation is a rare event as only 0.3% of tumors harbor a mutation in the encoding gene *CD274* (Huang et al., 2021). In addition, it was demonstrated that the targeting of the PD-1/PD-L1 axis, in absence of an appropriate number of T cells, may even lead tumor growth by reactivating AKT and PTEN signal cascade (Wang et al., 2020).

Anti-PD-L1 antibodies can be profitably used for the precise delivery of toxic payloads in a wide range of cancers that are positive for PD-L1, because the expression of PD-L1 was found in various solid malignancies, such as squamous cell carcinoma of the head and neck, melanoma and carcinomas of the brain, thyroid, thymus, esophagus, lung, breast, gastrointestinal tract, colorectum, liver, pancreas, kidney, adrenal cortex, bladder, urothelium, ovary, skin and others (Wang et al., 2016).

For this purpose, antibodies can be linked to pharmacologically active molecules, i.e., anti-cancer drugs, enzymes, hormones, toxins (mainly from plants or bacteria) and radionuclides. This approach allows to develop immunoconjugates also known as antibody-drug conjugates (ADCs), which have been evaluated in numerous preclinical studies and clinical trials (Tong et al., 2021). Immunoconjugates are usually composed by three portions: a carrier molecule (mainly a monoclonal antibody), a pharmacologically active molecule and a linker. After binding to the targeted antigen, the immunoconjugate is internalized and the active payload can exert its pharmacological effect. Several molecules have been considered for antibody–drug conjugate production; the most used agents act on DNA, such as doxorubicin, or on tubulin, blocking its depolymerization, thus determining cell-cycle arrest into G2/M phase (*i.e.*, monomethyl auristatin E, MMAE) (Hoffmann et al., 2017). Plant and bacteria toxins can be used in immunoconjugate construction, providing the so-called immunotoxins (Polito et al., 2016). Over the years, many immunotoxins have been constructed and tested in preclinical and clinical studies in different cancer models, showing promising results both in hematologic and solid tumors (Weidle et al., 2014; Polito et al., 2021). Radionuclides represent another type of payload used in targeted therapy. In this case, the antibody is radiolabeled with a radioisotope to obtain a radioimmunoconjugate (Steiner and Neri, 2011). Some human enzymes, such as carboxypeptidase, xanthine oxidoreductase, alkaline phosphatase, etc., and some hormones, such as melanocyte-stimulating hormone, have been also used as active portion of conjugates. The advantage of using these molecules consists in the possibility to produce, through genetic engineering, human-derived, and therefore non-immunogenic, payloads (Melton and Sherwood, 1996; Battelli et al., 2005). However, the scarcity of papers and the few encouraging results obtained with enzymes and hormones have discouraged most researchers from continuing to use these molecules for immunoconjugate construction.

## PD-L1 overview

PD-L1, belonging to the B7 family, is also known as B7H1 or CD274. It is a 33-kDa type 1 transmembrane glycoprotein containing 290 amino acids with IgV and IgC domains in its extracellular region. PD-L1 is expressed on a variety of normal cells, including epithelial and antigen presenting cells (APCs) (Burr et al., 2017; Mamessier et al., 2018). PD-L1 natural ligand is PD-1, also known as CD279, which is expressed on the surface of T and B lymphocytes and of myeloid cells. PD-1 binds to PD-L1 with a stoichiometry ratio of 1:1 with an affinity of 8.2 ± 0.1 μM, as measured by surface plasmon resonance (Zak et al., 2017). After PD-1/PD-L1 binding, the immune-related cell receives a negative modulation signal that avoids activation, maintaining systemic self-tolerance (Freeman et al., 2000). Cancer cells overexpress PD-L1 molecules on their membranes, thus leading T cell depletion and immune escape. Therefore, PD-L1 promotes proliferation and survival of cancer cells by binding to its receptor (Dong et al., 2018). As a consequence, the overactivation of PD-L1/PD-1 axis results in a poor cancer patient survival rate (Ahmadzadeh et al., 2009).

Currently, three anti-PD-L1 monoclonal antibodies (mAbs) have been approved by the FDA, namely, atezolizumab (Tecentriq) (Markham, 2016), durvalumab (Imfinzi) (Syed, 2017) and avelumab (Bavencio) (Kim, 2017).

The efficacy of immunoconjugate-based immunotherapy depends on four main parameters: the target antigen, the antibody, the cytotoxic payload and the linker between antibody and payload (Nejadmoghaddam et al., 2019). The ability of the antibody to induce the internalization of the complex assumes a relevant importance, mainly in the case of drugs or toxins that need to reach their intracellular target to damage the cell. Post translational modifications regulate different functions of transmembrane proteins, including their internalization. PD-L1 is exclusively N-glycosylated at N35, N192, N200 and N219 residues in cancer cells and those modifications have been demonstrated to stabilize the protein, preventing its interaction with glycogensynthase kinase 3β (GSK3β) and thus protecting PD-L1 from phosphorylation-dependent proteasome degradation (Li et al., 2016). Glycosylation of PD-L1 seems to be important for antibody-mediated receptor internalization as only antibodies that recognize the glycosylation sites N192 and N200 of PD-L1, but not those that are non-specific (IgG isotype control) or specific against the glycosylation site N35, induce PD-L1 internalization (Li et al., 2018). However, anti-PD-L1 mAbs atezolizumab, durvalumab and avelumab have been demonstrated to induce, at different levels, PD-L1 internalization, as described hereinafter, despite they do not interact with any of N35, N192, N200 and N219 glycosylated sites. Moreover, it was reported that PD-L1 glycosylation does not affect the binding of such mAbs (Lee et al., 2017).

Differently to the most of other B7 family members that are present as dimers in solution and in crystalline state, either alone or bound to their ligand, it is unclear whether the PD-L1 dimeric form exists in cells. In fact, crystallization studies on PD-L1 showed that its homodimer is not particularly prompt to form in solution, suggesting absence of PD-L1 dimeric form on the cell membrane (Chen et al., 2010). However, PD-L1 dimerization may be artificially induced by specific drugs that act as molecular bridges between two PD-L1 molecules. Atezolizumab, durvalumab and avelumab do not induce nor stabilize PD-L1 homodimers (Bailly and Vergoten, 2020).

## Atezolizumab

Atezolizumab, (Tecentriq, Genentech, Inc.), formerly known as MPDL3280A, is a fully human IgG1 antibody directed to PD-L1, indicated for the treatment of urothelial carcinoma (UC), non-small cell lung cancer (NSCLC), triple-negative breast cancer (TNBC), small cell lung cancer (SCLC), hepatocellular carcinoma (HCC) and melanoma.

Structure analysis revealed that both heavy chain variable domain (VH) and light chain variable domain (VL) of atezolizumab interact with PD-L1. Also, the atezolizumab VH rather than the VL domains play a dominant role in antigen binding. In particular, all three complementarity-determining region (CDR) loops of heavy chain of atezolizumab are involved in the binding, while only the CDR3 loop of light chain interacts with PD-L1 (Zhang et al., 2017). Superimposing the structure of PD-L1/atezolizumab complex with the structure of full-length PD-L1 (PDB: 5JDR) or PD-1/PD-L1 complex (PDB: 4ZQK) shows that PD-L1 structure is not significantly affected during atezolizumab binding. From superimposing analysis, it is also evident that atezolizumab conflicts with PD-1, so these molecules cannot bind to PD-L1 at the same time. Therefore, atezolizumab blocks PD-1/PD-L1 interaction by competing with PD-1 for the same surface area on PD-L1 (Tan et al., 2018).

Atezolizumab is provided with a modified Fc domain (substitution at position 298 Asn to Ala) that impairs its binding to Fcγ receptors, thus preventing antibody-dependent cellular cytotoxicity (ADCC) and complement-dependent cytotoxicity (CDC) (Lu et al., 2015). This allows to minimize, *in vivo*, the eventual depletion of the subset of PD-L1 expressing tumor specific T cells or APC (Deng et al., 2016). The inconvenience of this modification is that the null FcγR also prevents atezolizumab-mediated ADCC against cancer cells. However, the blockade of the PD-L1/PD-1 pathway may interfere with cancer cell viability independently of the interaction with immune system cells; in fact, it has been reported that in osteosarcoma cells atezolizumab is also able to induce apoptosis, autophagy and activating JNK pathway (Liu et al., 2021).

In the literature, different affinity values for the complex atezolizumab/PD-L1 have been reported. These differences mainly depend on PD-L1 post translational modification or complexing with other proteins. For example, atezolizumab has shown a K_d_ of 0.19 nM for the dimeric form of PD-L1 and a K_d_ of 0.62 nM for the monomeric form of PD-L1 (Kulikov et al., 2021). In another work, a K_d_ of 1.75 nM was reported but no information about PD-L1 status was given (Chen et al., 2020). Regarding the binding affinity for the glycosylated (gPD-L1) or non-glycosylated (ngPD-L1) form of PD-L1, atezolizumab displayed a K_d_ of 4.7 nM for gPD-L1 and 17.8 nM for ngPD-L1 (Benicky et al., 2021). Therefore, atezolizumab is suitable for binding both the monomeric and the dimeric form of PD-L1, as well as gPD-L1 and ngPD-L1 forms. Nevertheless, monomeric gPD-L1 seems to be the prevalent form on the cancer cell membrane.

Atezolizumab has proved to induce PD-L1 internalization in PD-L1 positive cell lines, SK-MES, PC-9 and Calu-1 (lung cancers), MDA-MB-231 (triple negative breast cancer), MC-38 (murine colon adenocarcinoma), A431 (epidermoid carcinoma) and PD-L1 transfected CHO-K1 (Chinese hamster ovary). In particular, up to 40% and 60% of PD-L1 protein could be internalized after 2 h in MDA-MB-231 or CHO-K1 and PC-9 cells, respectively, whereas SK-MES lysosomes are positive for atezolizumab after 16 h of treatment. Antibody concentration had little effect on PD-L1 internalization on PD-L1 transfected CHO-K1 cells. Atezolizumab showed a better internalization in comparison to avelumab in MC-38, SK-MES and MDA-MB-231 cells (Xiao et al., 2021).

Meta-analysis studies demonstrated that atezolizumab has tolerable adverse effects (AEs) in neoplastic patients. Major common AEs involved fatigue, decreased appetite, nausea, diarrhea, pyrexia, pruritus, cough, peripheral edema and rash. The most common severe AEs were fatigue, anemia and dyspnea. The incidence of atezolizumab-related pneumonitis is low, with 1% overall occurrence and <1% being grade 3, with no grade 4 events. The overall atezolizumab-related death rate and AEs were much lower than chemotherapeutics-related death rate (Rittmeyer et al., 2017; Tie et al., 2019).

### Atezolizumab conjugates

Different immunoconjugates have been obtained linking atezolizumab to different payloads, such as drugs, photosensitizers and radionuclides; the latter were mainly used as PET probes for biodistribution studies.

In 2021, Xiao and co-workers produced an immunoconjugate linking atezolizumab to MMAE. Atezolizumab was compared to avelumab and the first was chosen for its better cell uptake. The affinity of the MMAE-atezolizumab immunoconjugate for PD-L1 was comparable to that of the mAb alone (1.1 nM vs. 0.9 nM, respectively). The *in vitro* cytotoxicity of MMAE-atezolizumab was evaluated in MDA-MB-231, PC-9 and A431 cell lines, which displayed a PD-L1 positivity of 96.7%, 75.9% and 83.1%, respectively. MMAE-atezolizumab reduced cell viability with half-maximal effective concentration (EC_50_) values of 10.33, 9.75 and 11.94 nM, respectively. After conjugation, atezolizumab conserved its immune-stimulatory activity *in vitro* blocking PD-L1/PD-1 axis, as confirmed by the significantly increased levels of IFN-γ produced by peripheral blood mononuclear cells (PBMCs). MMAE-atezolizumab also displayed anti-tumor activity *in vivo*, in a mouse model represented by PD-1-humanized mice and a PD-L1-genetically engineered MC38 mouse colon cancer cell line. At the concentration of 3 mg/kg, the immunoconjugate produced a significantly augmented anti-tumor effect compared to atezolizumab alone (tumor regression of 60% and 40%, respectively). No significant body weight changes among the groups were founded (Xiao et al., 2021).

A doxorubicin-atezolizumab immunoconjugate resulted more effective in killing triple negative breast cancer cell line MDA-MB-231 compared to unconjugated doxorubicin, showing an EC_50_ of 1.25 and 4 μM, respectively. But, considering atezolizumab, the cytotoxic gain obtained after doxorubicin conjugation is marginal; it became significant only after 72 h and only at the highest concentration tested (2.5 μM). The immunoconjugate maintained the ability to induce INF-γ production in a co-culture of MDA-MB-231 cells and activated RAW 264.7 tumor-derived macrophage cells, thus demonstrating to modulate positively immune cell activation (Sau et al., 2019).

The photosensitizer chlorin e6 was conjugated to atezolizumab for photodynamic therapy. Photodynamic therapy works by systemic injection of a photosensitizer (PS) followed by irradiation of the tumor site with a specific wavelength of light, leading the activation of the PS, which in turn generates cytotoxic reactive oxygen species (ROS), ultimately killing cancer cells. The chlorin e6-atezolizumab conjugate was internalized into the endosomal compartment and chlorin e6 activation, after light irradiation, was confirmed *in vitro* by ^1^O_2_ detection. The conjugate demonstrated anti-tumor efficacy in HCT-116 (colon cancer cell line) tumor-bearing mouse model showing a tumor mass reduction greater than 50% compared to the same conjugate treatment without light activation. No data about the conjugate adverse effects *in vivo* were reported (Pan et al., 2021).

The atezolizumab ability to bind cells in tumor mass was exploited by using it as PET probe after radiolabeling with ^64^Cu or ^89^Zr for patient stratification or evaluation of the tumor response to immune modulation therapies (Lesniak et al., 2016; Bensch et al., 2018). The uptake of ^64^Cu atezolizumab was investigated *in vitro* on PD-L1 transfected CHO-K1, PD-L1 positive cell lines MDA-MB-231, SUM149 (both triple negative breast cancer) and 4T1 (mouse triple negative breast cancer), resulting positively correlated to PD-L1 cell surface expression (CHO-K1 > MDA-MB231 > 4T1 > SUM149) (Lesniak et al., 2016). *In vivo*, a similar trend of ^64^Cu atezolizumab uptake was reported in tumors induced in immunocompromised mice with the previously *in vitro* tested cell lines.

^89^Zr atezolizumab uptake was evaluated *in vitro* in H292 (pulmonary mucoepidermoid carcinoma) and H358 (NSCLC) cell lines, in healthy PBMCs and in T cells. Experimental observations showed that tumor cells have a higher uptake of ^89^Zr atezolizumab than non-tumoral cells. ^89^Zr atezolizumab was also tested in 22 patients with locally advanced or metastatic bladder cancer (n = 9), NSCLC (n = 9) or TNBC (n = 4). ^89^Zr atezolizumab tumor uptake, expressed as maximum standardized uptake value, was generally high, but with differences among tumor types. Specifically, ^89^Zr atezolizumab uptake of TNBC was on average 50% lower than bladder cancer and the highest uptake was towards liver metastases, whereas the lowest towards lung metastases. ^89^Zr atezolizumab injection was safe, with only one related low-grade adverse event (Bensch et al., 2018).

Radiolabeled ^111^In-PD-L1-mAb and near-infrared (NIR) dye conjugated NIR-PD-L1-mAb imaging agents were developed using atezolizumab. The specificity of ^111^In-PD-L1-mAb and NIR-PD-L1-mAb was tested in cell lines and in tumors with varying levels of PD-L1 expression. The highest percentage of PD-L1 expression was observed in CHO-PDL1 (99%) and in NSCLC H2444 (95%) cells. In mice bearing subcutaneous and orthotopic tumors, it was observed specific and persistent high accumulation of signal intensity in PD-L1 positive tumors (CHO-PDL1, MDAMB231, H2444), but not in controls. These results demonstrated that ^111^In-PD-L1- and NIR-PD-L1-mAbs can detect graded levels of PD-L1 expression *in vivo*, in human tumor xenografts. Specificity of NIR-PD-L1-mAb indicates the potential for optical imaging of PD-L1 expression in tumors, in relevant pre-clinical as well as clinical settings (Chatterjee et al., 2016).

## Durvalumab

Durvalumab (Imfinzi, AstraZeneca AB), also known as MEDI4736, is a human IgG1 mAb directed to the PD-L1 molecule approved by the FDA for the treatment of locally advanced or metastatic urothelial cancer, as well as stage III NSCLC, in inoperable and non-responder patients.

Durvalumab, as atezolizumab, has a constant domain modified by including three-point mutations in the molecule, in order to reduce the ADCC response (Stewart et al., 2015), and similar PD-L1/PD-1 blockade mechanism (Tan et al., 2018). Crystal structure analysis of durvalumab against PD-L1 revealed that both VL and VH are involved in the interaction. Durvalumab uses all three CDRs from the VH (HCDR1, HCDR2 and HCDR3) and two from the VL (LCDR1 and LCDR3) to form contacts with PD-L1, leaving LCDR2 without any binding (Lee et al., 2017).

Durvalumab displays high affinity to PD-L1, similarly to atezolizumab, with a K_d_ of 0.667 nM (Tan et al., 2018; Chen et al., 2020). In another work, durvalumab showed a K_d_ of 0.2 nM toward gPD-L1 form and a K_d_ of 0.4 nM for ngPD-L1 (Benicky et al., 2021).

Durvalumab exhibited a manageable safety profile with pruritus, fatigue, decreased appetite, diarrhea, increased AST level, nausea and rash as the most common AEs associated with its administration. Reported grade >3 AEs were decreased appetite and increased AST (Yang et al., 2018). The frequency of pneumonitis of any grade occurred in 33.9% of patients and pneumonitis of grade 3 or 4 occurred in 3.4% whereas pneumonia of any grade and of grades 3 to 4 was 13.1% and 4.4%, respectively (Antonia et al., 2017).

### Durvalumab conjugates

The plant toxin CUS245C, a type 1 ribosome-inactivating protein isolated from *Cucurbita moschata,* was linked to durvalumab to obtain a PD-L1-specific immunotoxin (named D-CUS245C) through an artificial disulfide bond. The immunotoxin efficacy was evaluated *in vitro* on MDA-MB-231 and PD-L1/SPC-A-1, human lung carcinoma stably transfected with a PD-L1 cDNA, cell lines; the parental NC/SPC-A-1 cell line was used as negative control (PD-L1 negative). D-CUS245C showed a dose-dependent cytotoxicity on PD-L1 overexpressing cell lines, with IC_50_ (half-maximal inhibition of protein synthesis) values of 3.8 pM and 1.6 pM for PD-L1/SPC-A-1 and MDA-MB-231, respectively. D-CUS245C also showed anti-tumor effect in an animal model consisting of nude mice bearing PD-L1 positive human tumors. A tumor weight reduction was observed (55% and 67% reduction with immunotoxin dose of 0.4 and 0.8 mg/kg, respectively) compared to the untreated group. Also, the tumor volume in the group receiving 0.4 mg/kg of unconjugated toxin was significantly reduced (*p* < 0.01) with respect to control group, but tumor weight was not significantly reduced. In conclusion, although *in vitro* the D-CUS245C immunotoxin showed an IC_50_ about 10.000-fold lower than CUS245C, when the immunotoxin was administered *in vivo* it showed an efficacy in tumor mass reduction quite similar to CUS245C (Zhang et al., 2020).

A clinical PET imaging study was conducted with ^89^Zr-labeled durvalumab (Smit et al., 2022). The study was conceived to search a good predictive biomarker for the treatment outcome in patients with advanced-stage NSCLC treated with anti-PD-L1 mAbs. Up to now, PD-L1 expression was the only accepted biopsy-based biomarker, but with several weaknesses, such as tumor heterogeneity of PD-L1 expression, even within the tumor and between various tumor lesions of the same patient and false-negative results (Buttner et al., 2017; Bigras et al., 2018). The study demonstrated that ^89^Zr-durvalumab is safe and well-tolerated and no serious adverse effects were observed. Tumor lesions could be visualized and quantified. Uptake heterogeneity was observed within and between patients. High uptake was obtained in liver and spleen, where ^89^Zr-durvalumab binds to PD-L1 receptors on lymphocytes and dendritic cells. In bone marrow, the uptake was a little higher than in blood. Low uptake was seen in kidneys, lungs and brain.

## Avelumab

Avelumab (Bavencio, Merck Serono SA) is a PD-L1 blocking antibody, approved by FDA and indicated for the treatment of patients with metastatic Merkel cell carcinoma, of patients with advanced or metastatic urothelial carcinoma and, in combination with axitinib, of patients with advanced renal cell carcinoma (Masters et al., 2022). Unlike atezolizumab and durvalumab that were engineered to reduce the Fc-mediated effector functions, avelumab retains the potential to induce ADCC and CDC (Lee et al., 2019).

Crystal structure analysis of avelumab revealed that both VH and VL regions bind to PD-L1, by involving five of the six CDRs (all three CDR loops of VH and LCDR1 and LCDR3 loop of VL). Superimposition of the PD-L1/avelumab-scFv complex structure with PD-1/PD-L1 complex structure showed that the binding area of avelumab on PD-L1 overlapped with that of PD-1 (Liu et al., 2017), suggesting that avelumab competes with PD-1 for the binding to PD-L1.

Avelumab has shown a higher binding affinity to PD-L1 in comparison to atezolizumab and durvalumab with a K_d_ value of about 0.042 nM (Liu et al., 2017; Chen et al., 2020). In particular, avelumab displayed a large affinity for gPD-L1 form (K_d_ of 0.4 nM for gPD-L1 and 17.0 nM for ngPD-L1) (Benicky et al., 2021). Therefore, avelumab is more suitable for binding to gPD-L1 form. Avelumab resulted in lower endocytosis efficiency with respect to atezolizumab, as avelumab was endocytosed by less than 20% of PD-L1 positive MC-38, SK-MES and MDA-MB-231 cells. This confirmed that atezolizumab is more suitable for immunoconjugate design (Xiao et al., 2021).

Avelumab administered as monotherapy is associated with high objective response rate and low AEs. Overall incidence of AEs was over 70%; however, they were generally low-grade and manageable and included infusion-related reaction, fatigue, nausea, diarrhea, decrease appetite, asthenia and rash. High-grade AEs were ALT and AST increase, lipase-level increase, anemia, infusion-related reaction, fatigue and gamma-glutamyl transferase increase. The frequencies of high-grade AEs were relatively low with no sign of increased toxicity due to the ADCC (Zhao et al., 2021). The odds to develop pneumonitis after avelumab administration was rare (Gulley et al., 2017), as well as the onset of grade 4 events or treatment-related deaths (Kaufman et al., 2016).

### Avelumab conjugates

Avelumab was conjugated to the photo-absorber IR700DX and its efficacy was investigated both *in vivo* and *in vitro*, in a PD-L1 expressing cell line, H441 (papillary adenocarcinoma of lung). The conjugate was internalized within 6 h of incubation in H441 cell line and showed rapid necrotic cell death after NIR exposure. *In vivo*, avelumab-IR700 showed high tumor accumulation and induced a significant tumor reduction and survival improvement after NIR treatment. No detectable signs of skin necrosis or toxicity attributable to the photo-absorber IR700DX were reported in any group. The conjugation of IR700DX to avelumab minimally altered the pharmacokinetics of the antibody due to the small size and hydrophilic nature of the photo-absorber (Nagaya et al., 2017).

An immuno-PET imaging study was conducted with avelumab labeled with the radionuclide zirconium-89. ^89^Zr-DFO-PD-L1 mAb was synthesized using avelumab conjugated to desferrioxamine. Binding and biodistribution studies were carried out *in vitro*, in MDA-MB231 cell line, and *in vivo*, in MDA-MB231 xenograft mouse models. *In vitro*, the mAb displayed high-affinity binding to PD-L1 and was able to detect also moderate PD-L1 expression levels. *In vivo*, tissue uptake of ^89^Zr-DFO-PD-L1 was correlated with PD-L1 expression levels and increased with escalating doses of avelumab. These results indicate that ^89^Zr-DFO-PD-L1 can be considered a potential candidate as a PET imaging agent to select patients for PD-L1-targeted immunotherapy and to monitor treatment response (Jagoda et al., 2019).

NDPMSH-αPD-L1 is a bispecific antibody, obtained by conjugating an analog of melanocyte stimulating hormone (MSH) to the anti-PD-L1 avelumab. NDPMSH-αPD-L1 maintains binding affinity to both melanocortin-1 receptor (MC1R) and PD-L1 on EK293-MC1R (melanoma) cells and showed thermal stability, serum stability and pharmacokinetic properties similar to the parental anti-PD-L1 mAb. The conjugate displayed a significant tumor growth inhibition (on day 23) *in vivo* in a syngeneic B16-SIY melanoma mouse model at 5 mg/kg dose, compared with the parental mAb. In 80% of treated mice, tumor size was under 500 mm^3^, significantly lower of that measured for saline- and avelumab-treated mice, for which tumor size was about 1500 mm^3^ and 2000 mm^3^, respectively. Analysis of tumor infiltrating lymphocytes revealed an increase of infiltrated T cells in the tumor microenvironment, suggesting that the strategy of using a tumor-targeted PD-L1-blocking bispecific antibody can enhance anti-tumor activity of anti-PD-L1 therapy alone (Zhang et al., 2019).

## Conjugates with other anti-PD-L1 antibodies

In addition to atezolizumab, durvalumab and avelumab, other anti-PD-L1 antibodies have been developed to obtain efficient carriers for delivering payloads inside tumor cells. The most used is the anti-PD-L1 mAb clone 10F.9G2 (IgG2b), which was obtained by immunizing Lewis rats with murine PD-L1 cDNA and murine PD-L1 CHO transfectants (Paterson et al., 2011).

The anti-PD-L1 mAb clone 10F.9G2 was used with the aim to elicit *in vivo* specific CD8^+^ cytotoxic T lymphocyte (CTL) response toward tumor. For this purpose, the mAb was covalently conjugated to the long E7 peptide from HPV-16. Once internalized by tumor cells, the E7 peptide is processed and exposed on the membrane in association to MHC I, thus activating immune response by CTLs against tumor cells. After administration to C57BL/6 mice bearing TC-1 (lung epithelial) cells, the conjugate generated a higher number of systemic and tumor-infiltrating E7-specific CTLs than those generated by E7 and 10F.9G2 administered either separately or in combination. Nevertheless, only the combination therapy with cisplatin led to stable, long-term control of cancer progression, thus demonstrating that the damage-associated molecular patterns (DAMPs) from dying tumor cells had a critical adjuvant effect after chemotherapy. In fact, only this therapeutic regimen was able to bring about a complete remission of the tumor after 29 days from inoculation and a survival of about 40% at the end of the experiment (Lee et al., 2021a).

The 10F.9G2 clone was conjugated to the NIR molecule IR700 (PD-L1-IR700) for photoimmunotherapy of ovarian cancer. The cytotoxicity of PD-L1-IR700 was tested *in vitro* on RAW 264.7 tumor-associated macrophage cell line and on two ovarian cancer cell lines, ID8-defb29-VEGF and MOSE. All the tested cell lines displayed a low or absent basal expression of PD-L1 that was induced by IFN-γ treatment. On ovarian cancer cells, the efficacy of treatment with PD-L1-IR700 was significantly higher after induction of PD-L1, whereas RAW 264.7 cells were sensible to PD-L1-IR700 treatment also in the absence of IFN-γ. *In vivo*, the conjugate was tested on PD-L1 positive orthotopic ovarian tumors. Tumor progression was reduced in the group of mice treated with the conjugate compared to control groups, which displayed at least two-fold bigger tumor mass. A lethal toxicity in eleven out of twenty (55%) tumor-bearing mice was detected within 24-hour post-treatment. Anti-PD-L1 antibody alone or IgG-IR700 and IR700-treated group did not caused toxicity. Subsequent investigations detected lung and kidney damage suggesting an involvement of neutrophil accumulation within the lungs (Jin et al., 2022).

IR700 was also conjugated to F(ab′)_2_ fragment of 10F.9G2; luciferase-expressing MC38 (murine colon cancer cells), LL/2 (murine Lewis lung carcinoma cells), and TRAMP-C2 (murine prostate cancer cell) cells were used to induce tumor in C57BL/6 mice. Quantification of luciferase activity revealed a 4–5-fold decrease in relative light units in the group of animals treated with the conjugate at 3 days after treatment, whereas control groups showed a gradual increase in relative light units, which correlate to the tumor growth. Consistent with the bioluminescence imaging results, tumor volumes in the group treated with the conjugate were 15–20 times lower than those in control groups. Mouse survival was significantly prolonged in the NIR-treated groups compared to control groups. One mouse over nine displayed a complete response and was disease-free. An advantageous and significant decrease in the number of CD11b^+^ Gr1^+^ myeloid-derived suppressor cells and activation of CTL and NK cells were reported (Taki et al., 2021).

The 10F.9G2 mAb was radiolabeled with ^89^Zr. The radioimmunoconjugate exhibited high target specificity and excellent binding to PD-L1–overexpressing CT26/PD-L1 (mouse colon cancer) cells compared to PD-L1 weakly expressing CT26 cells. *In vivo*, PET image analysis revealed that the conjugate accumulated in CT26/PD-L1 cells 3.9-fold more than in CT26 cells. *In vitro*, gemcitabine co-treatment increased ^89^Zr-10F.9G2 binding to 145.4% ± 7.8% of controls. In CT26 tumor bearing mice, gemcitabine enhanced ^89^Zr-anti-PD-L1 tumor uptake from 1.56 ± 0.48 % to 6.24 ± 0.37 % of the injected dose/g (Jung et al., 2021). In another work, the F(ab’)_2_ fragment of 10F.9G2 clone was radiolabeled with ^89^Zr. The conjugate maintained the same target specificity of the antibody fragment in B16F10 cells (murine melanoma). *In vivo*, PET image analysis showed earlier and higher tumor uptake than ^89^Zr-labeled full antibody (5.5 times more at 2- hour post injection) in B16F10-bearing mice (Bridgwater et al., 2020).

In a recent study, 10F.9G2 was conjugated to 10-nm lipid-coated superparamagnetic iron oxide (SPIO) nanoparticles (PD-L1-SPIO nanoparticles). The conjugates exhibited specific binding and internalization in PD-L1-expressing mouse glioblastoma cell line (GL261 cells). *In vivo* magnetic resonance imaging demonstrated that PD-L1-SPIO can detect PD-L1 expression in intra-tumoral and peritumoral regions in temozolomide-resistant glioblastoma bearing mice (Lee et al., 2021b). Gold nanoparticles (AuNPs) exhibit characteristic surface plasma resonance absorption in the NIR region and are promising agents for cancer photothermal therapy. This approach is based on the conversion of NIR light energy into heat and the generation of ROS, which can be utilized to ablate tumors. Therapeutic agents can also be coated onto the surface of AuNPs. In fact, the large surface area-to-volume ratio of AuNPs enables their surface to be coated with hundreds of drug molecules. This approach allows the drug and heat to be delivered specifically and simultaneously to tumor microenvironments.

For this purpose, doxorubicin and 10F.9G2 were conjugated to AuNPs (PD-L1-AuNP-DOX) for the targeted chemo-photothermal therapy of colorectal cancer. The conjugate evidenced cytotoxic effect by inducing apoptosis in CT-26 (murine colorectal cancer) cells with IC_50_ value of 0.25 μg/ml, that was significantly lower than IC_50_ values of doxorubicin alone (0.5 μg/ml). In the presence of NIR irradiation, PD-L1-AuNP-DOX significantly suppressed CT-26 cell proliferation by enhancing ROS generation (Emami et al., 2019).

Using a similar approach, AuNPs containing a human anti-PD-L1 mAb (unspecified) and the anti-cancer drug docetaxel (DOC) were constructed by copolymerization technique between polyethylene-glycol (PEG) and poly-ε-caprolactone (PCL). The DOC-PEG-PCL-mAb NPs conjugate showed high cellular uptake in MKN45 (gastric cancer) cell line. *In vitro*, cytotoxicity experiments on MGC803, MKN45 and HGC27 gastric carcinoma cell lines showed that this conjugate provided a mortality rate up to 65% that was slightly, but significantly, higher than control system represented by the same construct linked to a generic IgG. In addition, DOC-PEG-PCL-mAb NPs induced cell apoptosis and enhanced G2-M arrest in cancer cells, indicating the inhibition of microtubule synthesis (Xu et al., 2018).

AuNPs conjugated with murine anti-PD-L1 unspecified antibody (PD-L1-AuNP) showed cytotoxic effect on SCC-25 cells (oral squamous cell carcinoma) with respect to HaCaT (human keratinocytes) control cells. The treatment of SCC-25 cells with PD-L1-AuNP conjugates decreased the cell viability to 58% and 54% of that of untreated control, 24 and 48 h after treatment respectively. Moreover, PD-L1-AuNP-treated SCC-25 cells showed apoptotic changes, such as the cleavage of caspase-3, caspase-9 and poly (ADP-ribose) polymerase 1. PD-L1-AuNPs selectively bind to the SCC-25 cells promoting direct and immune cell-independent cancer cell apoptosis by reducing the PD-L1 protein levels, which in turn lead to the activation of the signal transducer and activator of transcription 3 and extracellular-signal-regulated kinase proteins (Choi et al., 2021).

In addition to AuNPs, immunoliposomes have been used for both therapeutic and diagnostic purposes. The anti-PD-L1 antibody (not specified in the paper) was conjugated to liposomes loaded with oxaliplatin, a third-generation platinum anti-cancer drug, and miRNA-130a, which is involved in the pathogenesis of different cancers. The anti-tumor effect of PD-L1 monoclonal antibody-conjugated miR-130a/oxaliplatin (PD–miOXNP) loaded immunoliposome was investigated *in vitro* in HGC27 (human gastric carcinoma) cell line. PD-miOXNP significantly decreased cell viability in a concentration-dependent manner by inducing apoptosis in 54% of cells, compared to untreated controls; treatment with oxaliplatin or liposomes loaded only with oxaliplatin and miRNA-130a induced apoptosis in about 10% and 30% of cells, respectively. *In vivo*, in HGC27-bearing BALB/c nude mice, PD-miOXNP showed significantly higher tumor growth inhibition (tumor volume of 450 mm^3^) than that observed in control group (tumor volume of 1600 mm^3^). The toxicity of the formulations was evaluated by regularly monitoring body weight until the study period ended (25 days). Mice treated with free oxaliplatin lost more than 10% of their body weight compared with the non-treated control mice, indicating a systemic toxicity of free oxaliplatin. In comparison, mice treated with immunoliposomes did not show any sign of body weight loss, indicating the safety of this delivery system (Wang et al., 2019).

The main characteristics and the therapeutic efficacy, *in vitro* and *in vivo*, of the conjugates obtained with the FDA approved mAbs atezolizumab, durvalumab and avelumab and with other anti-PD-L1 antibodies are listed in Table 1.

**TABLE 1:** *In vitro* and *in vivo* experiments carried out with anti-PD-L1 immunoconjugates.

Anti-PD-L1 antibody	Payload	*In vitro* experiments		*In vivo* studies		References
		Target cell line(s)	Dose and efficacy	Animal model	Dose and efficacy	
Atezolizumab	MMAE (drug)	MDA-MB-231 (breast cancer), PC 9 (lung adenocarcinoma), A431 (epidermoid carcinoma)	EC_50_ 10.33 nM, EC_50_ 9.75 nM, EC_50_ 11.94 nM	C57BL/6J-Pdcd1em1(hPDCD1)/Smoc mice	3 mg/kg i.p., 2 times per week, 6 times. TR 60% vs. 40% (atezolizumab alone)	Xiao et al. (2021)
	Doxorubicin (drug)	MDA-MB-231 (breast cancer)	EC_50_ 1.25 μM	n.a.	n.a.	Sau et al. (2019)
	Chlorin e6 (photosensitizer)	HCT-116 (colon carcinoma)	About 60% cell death at 10μM + 660 nm LED light at 20 mW cm^−2^ for 40 s	HCT-116-bearing nude mice	1 mg/kg i.v. + light irradiation 4 h post injection for 5 min. TR about 50% vs. control group	Pan et al. (2021)
Durvalumab	CUS245C (plant toxin)	MDA-MB-231(breast cancer), SPC-A-1 (lung cancer)	EC_50_ 1.6 pM, EC_50_ 3.8 pM	nu/nu genotype, BALB/c background	0.4–0.8 mg/kg i.v. on days 4, 8, 12 and 16. TR 55%–67% vs. controls	Zhang et al. (2020)
Avelumab	IR700DX (photosensitizer)	H441 (lung adenocarcinoma)	Over 80% cell death at 3 μg/ml + 32 J NIR light	H441-bearing homozygote athymic nude mice	100 μg i.v. + NIR light at 50 J/cm^2^ on day 1 and 100 J/cm^2^ on day 2. TR about 40% vs. control group	Nagaya et al. (2017)
	MSH (hormone)	HEK293 (human embryonic kidney)	n.a.	6B16-SIY-bearing C57BL/6 mice MC1R^+^/PD-L1^+^ (melanoma cells)	1 or 5 mg/kg, 4 i.p. injections every 3 days. Tumor size in 80% of mice under 500 mm^3^ at 5 mg/kg dose	Zhang et al. (2019)
10F.9G2	E7 peptide	n.a.	n.a.	TC-1-bearing C57BL/6 mice (lung carcinoma)	Cisplatin (5 mg/kg) + conjugate (210 μg/mouse), i.v. at days 7, 10 and 13. TR of about 100%; 40% of animals alive at the end of the experiments	Lee et al. (2021a)
	IR700 (photosensitizer)	ID8-defb29-VEGF, MOSE (ovarian cancer cells)	IFN-γ 10 ng/ml + Conjugate 20 μg/ml + NIR 32 J/cm^2^ significantly reduced cell viability	Orthotopic ID8-Defb29-VEGF- bearing mice	i.v. injection of 100 μg of PD-L1-IR700 plus irradiation at 200 J/cm2 TR of about 50%	Jin et al. (2022)
	Doxorubicin with AuNPs	CT-26 (murine colorectal cancer)	EC_50_ 0.25 μg/ml with 3 min NIR at 2.5 W/cm^2^ (808 nm laser).	n.a.	n.a.	Emami et al. (2019)
F(ab′)_2_ 10F.9G2	IR700 (photosensitizer)	MC38-luc- (colon cancer), LL/2-luc (Lewis lung carcinoma), TRAMP-C2-luc (prostate cancer)	EC_50_10 μg/ml conjugate + NIR at 128 J/cm^2^	MC38-luc-, LL/2-luc-, TRAMP-C2-luc-bearing C57BL/6 mice	100 μg/mice conjugate i.v. + NIR at 75 J/cm^2^ at day 4. Tumor volume reduction of 15–20 times	Taki et al. (2021)
Not specified	AuNPs	SCC-25 cells (oral squamous cell carcinoma)	Cell viability of 58% (24 h) and 54% (48 h) without irradiation	n.a.	n.a.	Choi et al. (2021)
Not specified	Docetaxel	MGC803, MKN45, HGC27 (gastric carcinoma)	Mortality rate ranging from 40% up to 65% after 48 h treatment at 120 ng/ml	n.a.	n.a.	Xu et al. (2018)
Not specified	Oxaliplatin + miRNA-130a in immunoliposomes	HGC27 (gastric carcinoma)	EC_50_ about 0.5 μg/ml Apoptosis in 54% of treated cells	HGC27-bearing BALB/c nude mice	Equivalent OXL concentration fixed at 7.5 mg/kg TR of about 72%	Wang et al. (2019)

Abbreviations: AuNPs, gold nanoparticles; EC50, effective concentration 50; i.v., intravenous; i.p., intraperitoneal; MSH, Melanocyte-stimulating hormone; NIR, near infrared; TR, tumor regression; n.a., not available.

## Discussion and conclusion

Since PD-1 was discovered, over 20 years ago, numerous experimental evidences have shown that PD-L1/PD-1 axis plays a crucial role in most cancers and its blockade represents a valid therapeutic approach in a wide range of solid and hematologic malignancies. This knowledge prompted the development of anti-PD-L1 antibodies, leading in 2016 to the approval of atezolizumab, followed in 2017 by the approval of durvalumab and avelumab. The development of anti-PD-L1 blocking mAbs is undoubtedly an important therapeutic opportunity and these mAbs have provided survival benefits for cancer patients. However, a significant number of patients do not respond to the anti-PD-L1 mAb therapy. In fact, it should be considered that often, in neoplastic patients, the reduced or absent infiltration of cytotoxic T cells and natural killer cells in the tumor microenvironment, or the presence of other immunosuppressive molecules, reduce the efficacy of the immunotherapy with anti-PD-L1 blocking antibodies.

The clinical administrations of atezolizumab, durvalumab and avelumab in patients affected by several neoplasm types have demonstrated that these ICIs are well tolerated. The occurrence of major common AEs involved fatigue, decreased appetite, nausea, diarrhea, pyrexia, pruritus, cough, peripheral edema and rash, which are usually manageable (Tie et al., 2019). At the moment, the data available in literature about the safety of anti-PD-L1 antibodies as carriers for toxic payload are still limited to few studies in mouse models (Nagaya et al., 2017; Bensch et al., 2018; Wang et al., 2019; Xiao et al., 2021).

The strategy to use anti-PD-L1 antibodies as carriers for toxic payloads may result in an improvement of their efficacy, independently of the immune system activation. A crucial requirement for the effective delivery of the payload toward its intracellular target is that the immunoconjugate complex is efficiently internalized and that the payload released inside the cell. Literature offers evidence that atezolizumab, durvalumab and avelumab are well internalized in PD-L1 positive cells, thus fulfilling the drug-targeting requirement. Among these antibodies, atezolizumab seems to be the best internalized. Nevertheless, all of them have been used for ADC strategy against cancer. It is noteworthy that also poorly internalized antibodies can be useful as immunoconjugates when the payload is represented by a radionuclide or when the immunoconjugate is used for tumor imaging purposes.

On one hand the results obtained linking payloads to anti-PD-L1 antibodies encourage researchers to develop more potent immunoconjugates to overcome the low response rate of unconjugated antibodies. On the other hand, the expression of PD-L1 on membrane surface of some normal cells may limit the opportunity to the use of anti-PD-L1 antibodies in ADC strategy. However, as above reported, PD-L1 has a higher expression and anti-PD-L1 mAbs have a better uptake in neoplastic than in normal cells. Therefore, before clinical use, it will be essential to establish for each anti-PD-L1 immunoconjugate its specific therapeutic window, i.e., the exact concentration range in which the immunoconjugate can produce the maximum antitumor effect, limiting any non-specific toxicity to tolerable levels.

In humans, tumors and metastasis uptake of radiolabeled atezolizumab and durvalumab administered to patients with bladder cancer, NSCLC and TNBC was also confirmed, suggesting that such patients, and probably also patients with other PD-L1 positive malignancies, would benefit from anti-PD-L1 immunoconjugates. The literature on anti-PD-L1 antibodies-based immunoconjugates is still limited but the reported studies have provided evidence that targeting PD-L1 with corresponding immunoconjugates may offer valid anti-cancer therapeutic tools. In addition to the three above cited anti-PD-L1 antibodies, already approved by FDA, other research grade anti-PD-L1 antibodies have been developed and successfully tested as payload carriers, although they are still not approved for clinical use. Besides classical antibody-payload conjugates, more recently, nanotechnology led to the production of nanogold particles and immunoliposomes, giving promising results both *in vitro* and *in vivo*.

The results obtained so far in preclinical studies with anti-PD-L1 immunoconjugates and the versatility of application in different tumor models encourage researchers worldwide to continue to improve these hybrid molecules to pursue the goal of translation from the bench to bedside.
